# The RNA-mediated estrogen receptor α interactome of hormone-dependent human breast cancer cell nuclei

**DOI:** 10.1038/s41597-019-0179-2

**Published:** 2019-09-16

**Authors:** Giovanni Nassa, Giorgio Giurato, Annamaria Salvati, Valerio Gigantino, Giovanni Pecoraro, Jessica Lamberti, Francesca Rizzo, Tuula A. Nyman, Roberta Tarallo, Alessandro Weisz

**Affiliations:** 1 Laboratory of Molecular Medicine and Genomics, Department of Medicine, Surgery and Dentistry “Scuola Medica Salernitana”, University of Salerno, 84081 Baronissi, SA Italy; 2 Genomix4Life srl, Department of Medicine, Surgery and Dentistry “Scuola Medica Salernitana”, University of Salerno, Baronissi, SA Italy; 3 Department of Immunology, Institute of Clinical Medicine, University of Oslo and Rikshospitalet Oslo, 0372 Oslo, Norway

**Keywords:** Proteomic analysis, Breast cancer

## Abstract

Estrogen Receptor alpha (ERα) is a ligand-inducible transcription factor that mediates estrogen signaling in hormone-responsive cells, where it controls key cellular functions by assembling in gene-regulatory multiprotein complexes. For this reason, interaction proteomics has been shown to represent a useful tool to investigate the molecular mechanisms underlying ERα action in target cells. RNAs have emerged as bridging molecules, involved in both assembly and activity of transcription regulatory protein complexes. By applying Tandem Affinity Purification (TAP) coupled to mass spectrometry (MS) before and after RNase digestion *in vitro*, we generated a dataset of nuclear ERα molecular partners whose association with the receptor involves RNAs. These data provide a useful resource to elucidate the combined role of nuclear RNAs and the proteins identified here in ERα signaling to the genome in breast cancer and other cell types.

## Abstract (summary)

**Table:** 

Design Type(s)	parallel group design • protein and RNA interaction identification objective • cell type comparison design
Measurement Type(s)	RNA-Protein Interaction • protein-protein interaction detection
Technology Type(s)	mass spectrometry
Factor Type(s)	experimental condition • biological replicate
Sample Characteristic(s)	MCF7 cell

Machine-accessible metadata file describing the reported data (ISA-Tab format)

## Background & Summary

**Fig. 1:**
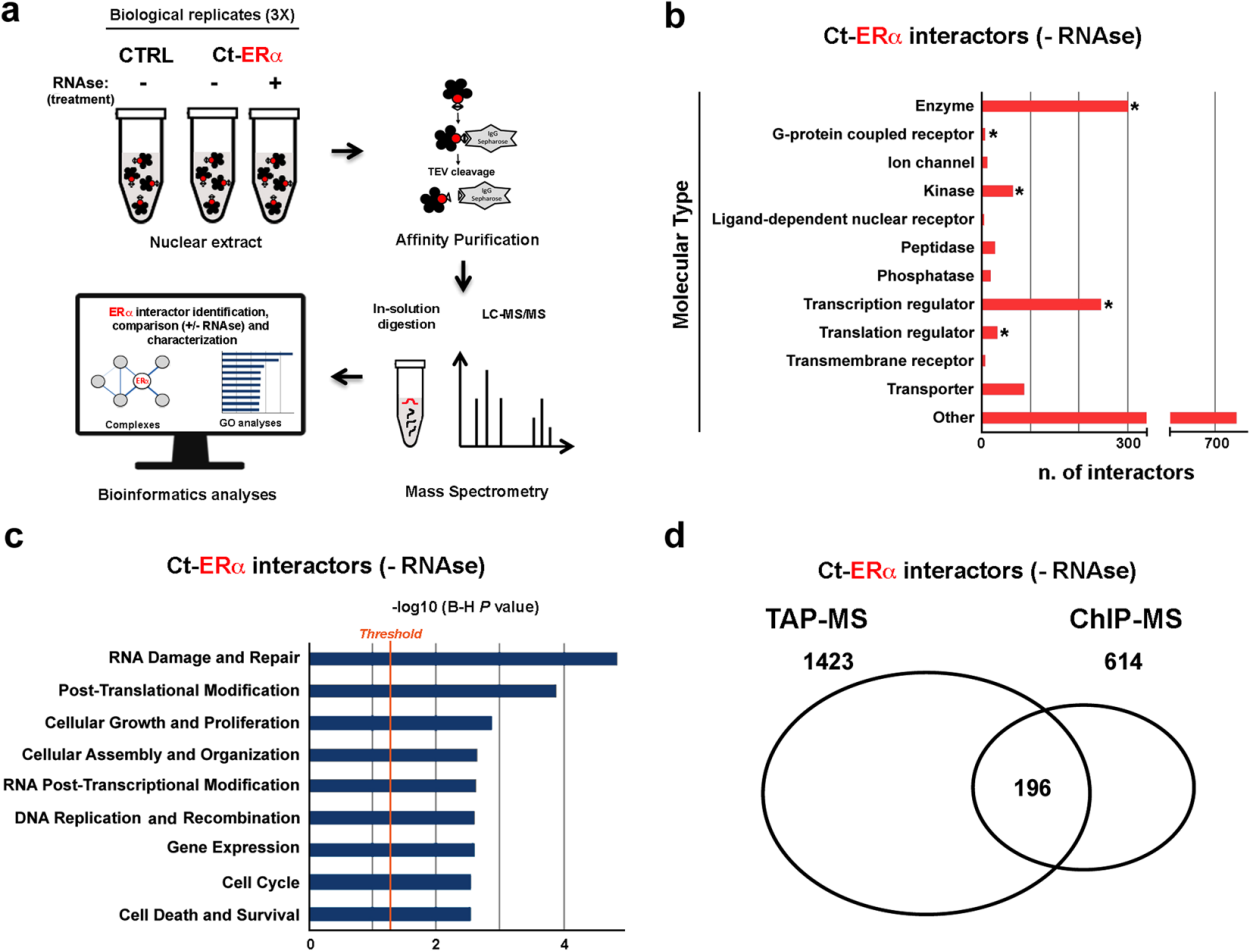
Characterization of ERα interactome. (**a)** Summary of the experimental workflow applied to generate the protein datasets. Ct-ERα: MCF7 cells stably expressing TAP-tag at the ERα C-terminal; CTRL: *wt* MCF7 cells. **(b)** Classification of ERα molecular partners; asterisks indicate statistically enriched molecule types (p < 0.01 hypergeometric test). **(c)** Functional enrichment analysis by IPA of ERα-associated proteins (B-H: Benjamini-Hochberg corrected p-value). **(d)** Venn diagram showing the overlap between ERα interactors identified here by Tandem affinity purification (TAP) and a dataset previously generated through Chromatin immunoprecipitation followed by mass spectrometry (ChIP-MS), described by *Nassa et al*.^8^.

The Estrogen Receptor alpha (ERα), a ligand-inducible transcription factor encoded by the ESR1 gene, is a key mediator of the estrogen signaling in hormone-responsive breast cancer (BC)^1^. This receptor subtype exerts a direct control on gene transcription machinery by binding chromatin cistromes^2^, where it assembles in large functional multiprotein complexes. These complexes comprise several molecular partners endowed with different functions, including co-regulators^3–5^ and epigenetic modulators^6–8^ that drive gene expression changes underlying BC development and progression^9^. Thus, the identification and characterization of the multiprotein complexes involved in the mechanism of action of ERα are crucial steps to understand the molecular bases of its signaling. Systematic application of interaction proteomics by coupling complexes purification with mass spectrometry analyses, represent an optimal experimental approach to gain such information. In the context of regulatory network assembly, it has emerged that RNAs of different nature may play a critical role as hinge of multiprotein complexes. Yang and colleagues^10^ revealed the importance of long non-coding RNAs in mediating androgen receptor function, acting as bridging molecules in the formation of functional protein complexes of this nuclear hormone receptor. On the other side, it is known that ERα has the ability to bind RNAs and that some of those are present within the receptor-mediated transcriptional complexes^11^. Thus, we assessed here the involvement of RNAs in mediating ERα-driven nuclear protein network assembly. To this aim, ERα-bound native proteins were purified from BC cell nuclei, before and after *in vitro* RNase treatment, and subjected to protein identification and quantification (Fig. 1a). In detail, Tandem Affinity Purification (TAP)^12–14^ followed by mass spectrometry was performed in parallel from Ct-ERα (MCF7 cells stably expressing ERα tagged with the TAP moiety at the C-terminus) and parental cells (CTRL: MCF7 parental line). This resulted in the initial identification of 1423 ERα protein partners^15^. Indeed, to avoid the inclusion of false positives and strengthen the specificity of our data, we defined ERα molecular partners only those proteins that were completely absent in the CTRLs purifications performed in parallel, according to the analytical steps detailed in the methods section.

The interactome identified, comprising among other enzymes, transcription regulators and kinases (Fig. 1b) (p-value < 0.05, hypergeometric test), defines molecular functions known to be involved in ERα cellular functions, such as cell growth and proliferation, gene expression, cell cycle and cell death and survival (Fig. 1c) (p-value < 0.05, Fisher’s test, Benjamini-Hochberg correction). The identified proteins include several known ERα molecular partners^12^, including a sizable fraction (32%, 196) recently shown to be part of chromatin-associated multiprotein complexes important for ERα activity in BC^8^ (Fig. 1d). Then, to understand whether and how RNA moieties can mediate the assembly or composition of these complexes, nuclear extracts were pre-treated by RNase digestion before TAP and MS analysis using the same experimental approach detailed in Fig. 1a. To assess the effectiveness of the enzymatic treatment, RNA was purified from an aliquot of each nuclear extract before and after RNase and analyzed by microfluidic electrophoresis as previously described^14,16^. In parallel, aliquots of each sample were kept from each TAP passage and analyzed by western blotting for the presence and integrity of ERα as detailed in the technical validation section. Molecular partners associated with the receptor even after RNase treatment were identified according to the same procedure described above, by comparing RNase+ and RNase− datasets, after filtering nonspecific interactors retrieved also in control purifications.

**Fig. 2:**
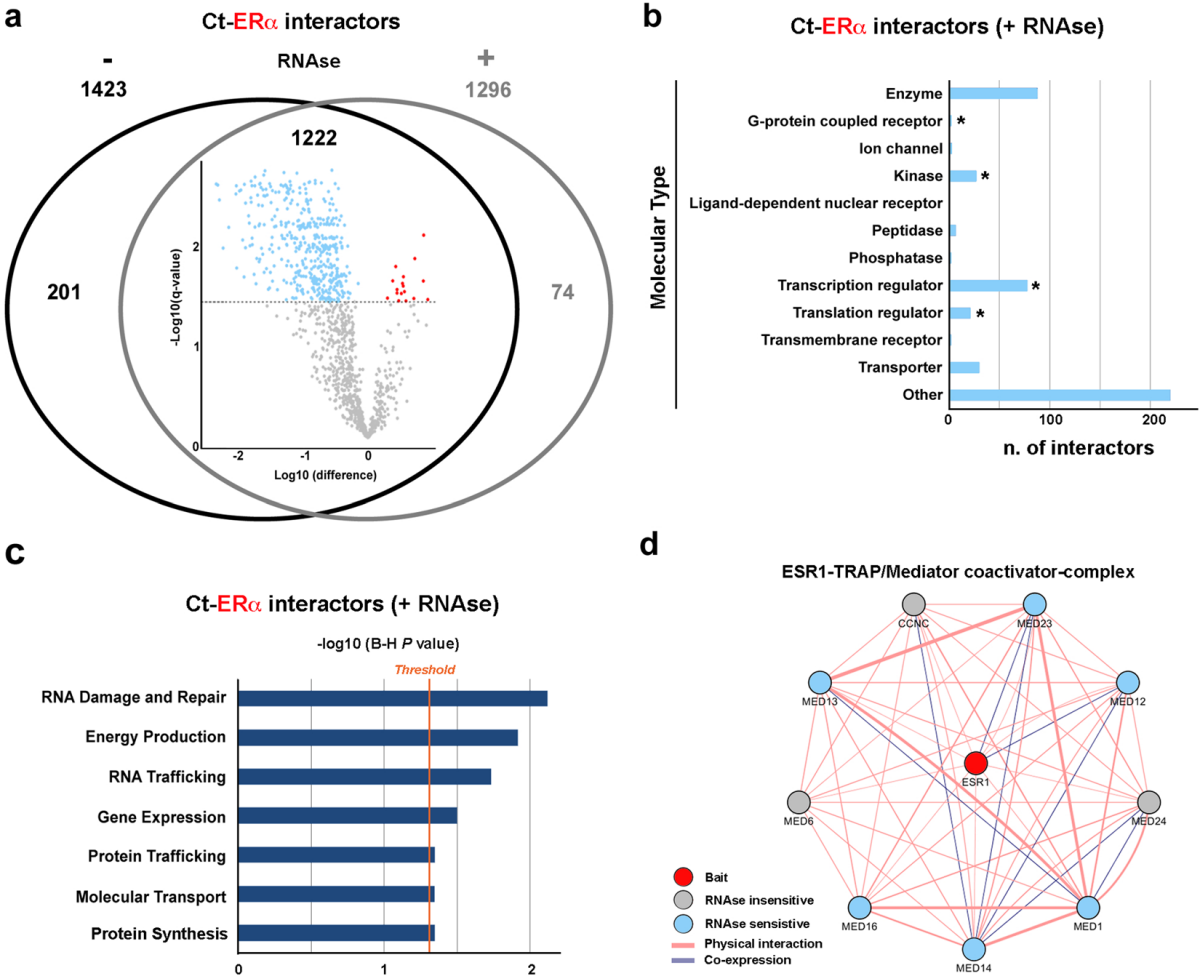
Analysis of ERα interactome changes upon RNase treatment. (**a)** Venn diagram showing the overlap between ERα molecular partners identified by Tandem affinity purification (TAP) before and after RNase treatment and Volcano plot summarizing quantitative changes of ERα-associated overlapping proteins upon treatment with RNase. Dotted line (threshold) represents the cut-off (q-value ≤ 0.05). **(b)** Classification of ERα molecular partners; asterisks indicate statistically significant molecule types (p < 0.01 hypergeometric test). **(c)** Functional enrichment analysis by IPA of ERα-associated proteins (B-H: Benjamini-Hochberg corrected p-value). **(d)** Network representation of the ESR1-TRAP/Mediator coactivator-complex; thickness of links (lines) among nodes (proteins) is proportional to the strength of the physical interaction. Information about co-expression, physical interactions and strength derive from GeneMANIA.

The dataset obtained results in 1296 proteins. By comparing ERα interactomes under the two experimental conditions, it turned out that they shared about 90% of the interactors (Fig. 2a). A quantitative approach was then applied, by using Perseus^17^, to identify proteins whose concentration was significantly reduced by RNase digestion, compared to untreated samples. The quantitative analyses were performed considering the 1222 ERα partners identified and quantified in both untreated (RNase−) and treated (RNase+) samples. Statistical analyses revealed that the concentration of about 35% of these proteins was significantly modulated by RNA depletion (q-value ≤ 0,05) (Volcano plot in Fig. 2a and in figshare^15^), indicating that RNAs are likely to mediate their association with the receptor in BC nuclei. ERα molecular partners affected by RNase treatment, meaning the interacting proteins whose concentration in the purified samples was affected by RNase pre-treatment (reported in the Volcano plot in light blue) comprise several enzymes, such as, transcription regulators and kinases (Fig. 2b) (p-value < 0.05, hypergeometric test). These factors are involved in key estrogen receptor functions in BC, including gene expression and protein synthesis regulation as shown in Fig. 2c. Complex analyses *via* g:Profiler^18^ was then used to gather information concerning ERα multiprotein complexes assembly^15^.

Considering the functions of the 1222 ERα partners, it turned out that they assemble in multiple complexes^15^, such as for example the Mediator complex, known to have a key role in estrogen receptor-mediated gene transcription, and the ESR1-TRAP/Mediator coactivator-complex (where ESR1 indicates the gene coding for the ERα protein). The latter, as shown as an example in Fig. 2d, includes several proteins whose association with the receptor was decreased by RNase treatment.

In conclusion, the RNA-dependent nuclear interactome reported here will be useful to investigate in greater detail the molecular mechanisms underlying ERα actions in BC cells, characterizing the RNA(s) involved and other key nodes of this regulatory network, toward identification of druggable targets against breast and other cancers where ERα plays a pivotal role.

## Methods

### ERα nuclear complexes purification

MCF7 cells stably expressing ERα fused with the TAP-tag at the C-terminus (Ct-ERα), to allow proper protein complexes purification, were generated as earlier detailed^13,19^. Ct-ERα and *wt* (CTRL) MCF7 cells (ATCC HTB-22), exponentially growing, were harvested by scraping in cold PBS and lysed as previously described^19^ in order to obtain nuclear extracts as reported by Giurato and co-workers^14^. To this aim, cell pellets were resuspended in 3 volumes of hypotonic buffer (20 mM HEPES pH 7.4, 5 mM NaF, 10 µM sodium molybdate, 0.1 mM EDTA, 1 mM PMSF and 1X protease inhibitors cocktail (Sigma Aldrich) and incubated on ice for 15 minutes. Cytosolic fraction was discarded after adding 0.5% Triton X-100 and spinning for 30 sec at 15000 × g at 4 °C. Nuclear pellets were then resuspended in 1 volume of nuclear lysis buffer (20 mM HEPES pH 7.4, 25% glycerol, 420 mM NaCl, 1.5 mM MgCl_2_, 0.2 mM EDTA, 1 mM PMSF and 1X protease inhibitors cocktail (Sigma Aldrich), incubated for 30 minutes at 4 °C under gentle shaking and centrifuged for 30 min at 15000 × g at 4 °C. Supernatants were then collected, diluted 1:3 with nuclear lysis buffer without NaCl, to restore the physiological saline concentration, and quantified.

For TAP procedure, IgG-Sepharose beads (GE Healthcare), pre-treated according to the manufacturer’s instructions and equilibrated in TEV buffer (50 mM Tris-HCl pH 8.0, 0.5 mM EDTA, 0.1% Triton X-100, 150 mM NaCl, 1 mM DTT), were added to nuclear protein extracts and incubated for 3 hours at 4 °C with gentle rotation, as described earlier^12,20^. 100 µg/ml RNaseA were added to the samples before binding, as already reported^14,16^ (see Table 1). After incubation, unbound proteins were discarded following centrifugation and the beads were thoroughly washed with 100xVol of IPP150 buffer (20 mM HEPES pH 7.5, 8% glycerol, 150 mM NaCl, 0.5 mM MgCl2, 0.1 mM EDTA, 0.1% Triton X-100) and equilibrated in 30xVol of TEV Buffer in Poly-Prep Chromatography columns (0.8 × 4 cm, Bio-Rad) at 4 °C. Then, 4xBeads Vol of Cleavage Buffer (TEV Buffer containing 1U/μl beads of TEV protease, Invitrogen) were added and two subsequent cleavage reactions were carried out for 2 hours and 30 minutes respectively at 16 °C with gentle shaking. The eluates were then collected, after sedimentation of beads still binding uncut and non-specific proteins. The same procedure was performed in parallel from Ct-ERα and *wt* MCF7 cells that served as negative control to identify nonspecifically isolated proteins to be discarded.

**Table 1:** Summary of the protocols and datasets used.

Sample name (cell line)	Protocol 1	Protocol 2	Protocol 3	Treatment	Data
MCF7 (CTRL)_1	Nuclear protein extracts	Tandem Affinity Purification	Nano LC-MS/MS	—	PRIDE PXD012630
MCF7 (CTRL)_2	Nuclear protein extracts	Tandem Affinity Purification	Nano LC-MS/MS	—	PRIDE PXD012630
MCF7 (CTRL)_3	Nuclear protein extracts	Tandem Affinity Purification	Nano LC-MS/MS	—	PRIDE PXD012630
Ct-ERα (sample)_1	Nuclear protein extracts	Tandem Affinity Purification	Nano LC-MS/MS	—	PRIDE PXD012630
Ct-ERα (sample)_2	Nuclear protein extracts	Tandem Affinity Purification	Nano LC-MS/MS	—	PRIDE PXD012630
Ct-ERα (sample)_3	Nuclear protein extracts	Tandem Affinity Purification	Nano LC-MS/MS	—	PRIDE PXD012630
Ct-ERα (sample)_1	Nuclear protein extracts	Tandem Affinity Purification	Nano LC-MS/MS	RNase A	PRIDE PXD012630
Ct-ERα (sample)_2	Nuclear protein extracts	Tandem Affinity Purification	Nano LC-MS/MS	RNase A	PRIDE PXD012630
Ct-ERα (sample)_3	Nuclear protein extracts	Tandem Affinity Purification	Nano LC-MS/MS	RNase A	PRIDE PXD012630

### Nano LC-MS/MS and data analysis

For mass spectrometry analyses, three biological replicates of TEV-eluted samples from control MCF-7 and from Ct-ERα cells before and after RNase treatment were analyzed. Protein extracts were precipitated with 10% TCA in acetone solution and and the proteins were reduced, alkylated and in-solution digested with trypsin (Promega) with the ProteaseMAX™ Surfactant (Promega) protocol according to manufacturers’ instructions. The resulting peptides were desalted and concentrated before mass spectrometry by the STAGE-TIP method using a C18 resin disk (3 M Empore). The peptides were eluted with 0.1% TFA/50% ACN, dried and solubilized in 7 μL 0.1% FA for mass spectrometry analysis. Each peptide mixture was analyzed on an Easy nLC1000 nano-LC system connected to a quadrupole Orbitrap mass spectrometer (QExactive, ThermoElectron, Bremen, Germany) equipped with a nanoelectrospray ion source (EasySpray/Thermo). For the liquid chromatography separation of the peptides an EasySpray column capillary of 25 cm bed length was employed. The flow rate was 300 nL/min, and the peptides were eluted with a 2–30% gradient of solvent B in 60 minutes. Solvent A was aqueous 0.1% formic acid and solvent B 100% acetonitrile/0.1% formic acid. The data-dependent acquisition automatically switched between MS and MS/MS mode. Survey full scan MS spectra were acquired from a mass-to-charge ratio (m/z) of 400 to 1,200 with the resolution R = 70,000 at m/z 200 after accumulation to a target of 3,000,000 ions in the C-trap. For MS/MS, the ten most abundant multiple-charged ions were selected for fragmentation in the high-energy collision dissociation (HCD) cell at a target value of 100,000 charges or maximum acquisition time of 100 ms. The MS/MS scans were collected at a resolution of 17,500. Target ions already selected for MS/MS were dynamically excluded for 30 seconds. The resulting MS raw files of control MCF-7 cells were submitted for protein identification using Proteome Discoverer (ver 2.1) software with the Mascot (ver 2.6.1) search engine. The search criteria for Mascot searches were: trypsin digestion with two missed cleavage allowed, Carbamidomethyl (C) as fixed modification and Acetyl (N-term), Gln- > pyro-Glu (N-term Q), Oxidation (M) as variable modifications. The parent mass tolerance was 10 ppm and MS/MS tolerance 0.1 Da. Database searches were done against the UniProt Human database (September 2018) and known contaminants provided by MaxQuant. All of the reported protein identifications were statistically significant (p < 0.05) in Mascot, and further filtered in ProteomeDiscoverer to report only high and medium protein FDR confidence identifications.

For the experiment performed in presence or absence of RNase the resulting MS raw files were submitted to the MaxQuant software (version 1.6.2.10)^21^ for protein identification and quantitation using the Andromeda search engine. MaxQuant search was done against the UniProt Human database (September 2018). Carbamidomethyl (C) was set as a fixed modification and protein N-acetylation and methionine oxidation were set as variable modifications. First search peptide tolerance of 20 ppm and main search error 4.5 ppm were used. Trypsin without proline restriction enzyme option was used, with two allowed miscleavages. The minimal unique + razor peptides number was set to 1, and the allowed FDR was 0.01 (1%) for peptide and protein identification.

Further analysis of the LC-MS/MS data followed two steps. Firstly, only proteins identified in the Ct-ERα samples (before and after RNase treatment) and not present in parental MCF7 cells (CTRL) were considered. In details, proteins were considered specifically associated with Ct-ERα before RNase treatment if they were present in at least two out of three biological replicates, but not after RNase treatment. The same approach was followed to consider proteins specifically associated with Ct-ERα after RNase. As commonly identified proteins we reported those present in at least two out of three replicate samples, in both experimental conditions tested. Moreover, known contaminants provided by MaxQuant and potential contaminants identified only in the Ct-ERα samples (e.g. Keratins, Immunoglobulins) were discarded and excluded from further analysis^15^. Statistical analysis was performed using Perseus software (version 1.6.1.3). To this aim, data (INTENSITY) was log10 transformed, filtered to include only proteins identified and quantified in at least two of the three replicates in at least one experimental group. Missing values were imputed with values representing a normal distribution with default settings in Perseus 1.6.1.3^17^. To find statistically significant differences between the two groups (RNase *vs* Sample) a T-test with a permutation-based approach was applied, with an FDR cut-off of 0.05. The lists of proteins whose association with the receptor changed after depletion of RNA have been deposited in figshare^15^. The mass spectrometry proteomics data have been deposited to the ProteomeXchange Consortium^22^ via the PRIDE^23^ partner repository with the dataset identifier PXD012630^24^ for interaction and quantitative proteomics datasets comprising CTRLs and Ct-ERα samples before and after RNase treatment. The protein interactions from this publication have been submitted to the IMEx (http://wwww.imexconsortium.org) consortium through IntAct^25^ and assigned the identifier IM-26954^26^.

### Functional annotation analyses

Functional annotation analysis was performed using Ingenuity Pathway Analysis (IPA, QIAGEN, Redwood City, www.qiagen.com/ingenuity) setting the following parameters:

- Reference set: Ingenuity Knowledge Base
- Relationship to include: Direct and Indirect
- Filter: Consider only molecules and/or relationship where (species:Human) AND (confidence: Experimentally Observed)

Only functions showing a Benjamini Hochberg (B-H) pvalue ≤ 0.05 were considered.

Molecular type information was retrieved from IPA and the pvalue associated to over-enrichment of each molecular category was computed using hypergeometric test. The information for protein categories was retrieved from UniProt.

Over-representation analysis on protein complexes was performed using g:Profiler^18^, in order to gather information concerning ERα multiprotein complexes assembly, setting the following parameters:

- Organism: “Homo Sapiens”
- All results: yes
- Statistical domain scope: “Only annotated genes”
- Significance threshold: “g:SCS threshold”
- User threshold: “0.05”
- Numeric ID treated as: “ENTREZGENE_ACC”
- Data sources: “CORUM”

Only complexes showing an adjusted-pvalue ≤ 0.05 were considered. Network was created with Cytoscape v. 3.7.1^27^, using the GeneMANIA^28^ application and considering only “Physical Interaction” and “Co-expression”. Physical interactions, co-expression, and strength information derive from GeneMANIA internal database.

## Data Records

Data records are available to be downloaded from Figshare archive, in which data were deposited including uncropped Western blots of the TAP procedures^15^.

The mass spectrometry proteomics data have been deposited to the ProteomeXchange Consortium via the PRIDE partner repository with the following dataset identifier: PXD012630 for interaction and quantitative proteomics datasets comprising CTRLs and Ct-ERα samples before and after RNase treatment^24^.

The Protein-Protein interactions have been submitted to the IMEx (www.imexconsortium.org) consortium through IntAct^25^ and assigned the identifier IM-26954^26^.

## Technical Validation

To ensure quality and robustness of the data presented here, our datasets were generated from three, independent biological replicates by using cell cultures processed independently from authenticated and Mycoplasma-free MCF7. This cell line represents a widely used model of Luminal A BC that have been demonstrated to have fundamentally altered the course of breast cancer research and have contributed to the improvement of patient’s outcomes^29^, suggesting that it is unlikely that the results obtained here are all specific only of the cell line used.

**Fig. 3:**
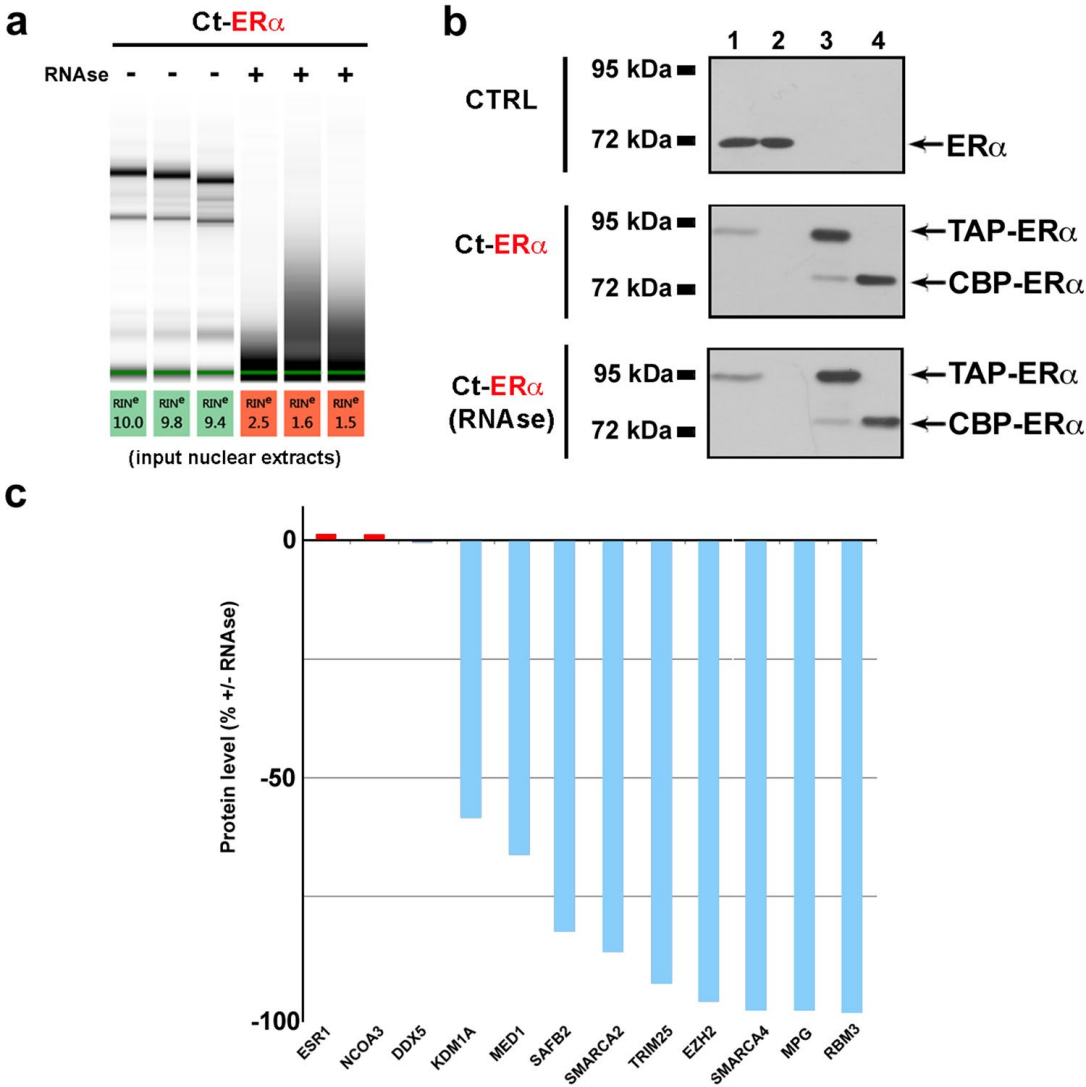
Quality controls of the experimental procedure. (**a)** Electrophoretic analysis of RNA extracted from nuclear extracts (starting material) before and after RNase treatment. **(b)** Representative Western Blot (one of the three biological replicates of the study) of the different steps of the Tandem Affinity Purification protocol in wt MCF-7 (CTRL, up) and Ct-ERα cells before (middle) and after (down) RNase treatment. The presence of the indicated proteins has been evaluated in different fractions as described: lanes 1 and 2, Crude nuclear extracts before and after IgG-Sepharose binding respectively; Lanes 3, IgG-Sepharose-bound proteins before TEV elution; Lanes 4 (TEV elution), proteins eluted from IgG-Sepharose. **(c)** Bar plot showing protein levels (%) of ERα and some of its interactors (+vs − RNase). Unchanged proteins are reported in gray, decreased proteins in blue.

Each TAP biological replicate was performed independently and both controls and samples were analyzed in parallel. The effect of the RNase treatment and efficiency of the affinity purification procedure were rigorously assessed as described previously^14,16^ and reported in Fig. 3a,b respectively. Correlation between the biological triplicate purifications (about 90%) was verified by analyzing log10 intensities with R version 3.5.2 (see code availability 7), applying the function *co*r from R-package sats v3.5.2 with Pearson correlation. Additionally, principal component analysis (PCA) highlighted the differences between the two treatments (−/+ RNase treatment) and the minimal variation among biological replicates of each group. Lastly, to exclude that the observed proteome changes are due to a reduction of receptor concentration in the crude nuclear extracts as a consequence of RNase treatment, ERα (ESR1) median intensities of the three biological replicates before and after RNase treatment were compared (Fig. 3c). This and other laboratories investigated previously ERα interactome in breast cancer cells using different approaches and under different experimental conditions^8,12,13,30,31^.

The dataset described here includes, also, a sizeable fraction of previously reported ERα interactors from BioGRID database (about 30%). In addition, it reveals that some of the best known key mediators of estrogen signaling to the genome, such as NCOA3^32^ and DDX5^33^, remain associated with ERα after RNase treatment. Others, representing about 10% (according to the ESR1/ERα BioGRID known interactions) of the 201 proteins whose concentration resulted to be modulate by RNase treatment, and including KDM1A^34^, MED1^35^, SAFB2^36^, SMARCA2/4^5^, TRIM25^37^, EZH2^38^, MPG^39^ and RBM3^40^, decrease strongly upon RNA removal, suggesting that their interaction with the receptor might be mediated by RNAs^41^ (Fig. 3c).

## ISA-Tab metadata file

Download metadata file

## Data Availability

The following software and versions were used for quality control and data analysis: 1. For protein identification from raw MS data, Proteome Discoverer software (Thermo) version 2.1 was used: https://tools.thermofisher.com/ with the Mascot 2.6.1 search engine. 2. For quantitative data analysis, MaxQuant software version 1.6.2.10 with the Andromeda search engine was used: http://www.coxdocs.org/docu.php?id=maxquant:start 3. For statistical proteomics analysis, Perseus software version 1.6.1.3 was used: (http://www.perseus-framework.org) 4. The UniProt human database was used (September 2018) as database for protein searches: https://www.uniprot.org/ 5. Functional analysis was performed with IPA software version 2.4: http://www.qiagenbioinformatics.com/products/ingenuity-pathway-analysis 6. Complexes analysis was performed using g:Profiler: https://biit.cs.ut.ee/gprofiler/gost 7. Statistical analyses were performed using R 3.5.2: www.r-project.org 8. The R script and the data used to create volcano plot are available at the Github repository https://github.com/LabMedMolGE/VolcanoPlot.
